# The recent history and near future of digital health in the field of behavioral medicine: an update on progress from 2019 to 2024

**DOI:** 10.1007/s10865-024-00526-x

**Published:** 2024-10-28

**Authors:** Danielle Arigo, Danielle E. Jake-Schoffman, Sherry L. Pagoto

**Affiliations:** 1 Department of Psychology, Rowan University, Glassboro, NJ USA; 2 Department of Family Medicine, Rowan-Virtua School of Osteopathic Medicine, Stratford, NJ USA; 3 Department of Biomedical Sciences, Cooper Medical School of Rowan University, Camden, NJ USA; 4 Department of Health Education & Behavior, University of Florida, Gainesville, FL USA; 5 Department of Allied Health Sciences, Center for mHealth and Social Media, Institute for Collaboration in Health, Interventions, and Policy, University of Connecticut, Storrs, CT USA

**Keywords:** Digital health, Mobile applications, Behavior change intervention, Artificial intelligence, Wearable technology

## Abstract

The field of behavioral medicine has a long and successful history of leveraging digital health tools to promote health behavior change. Our 2019 summary of the history and future of digital health in behavioral medicine (Arigo in J Behav Med 8: 67–83, 2019) was one of the most highly cited articles in the *Journal of Behavioral Medicine* from 2010 to 2020; here, we provide an update on the opportunities and challenges we identified in 2019. We address the impact of the COVID-19 pandemic on behavioral medicine research and practice and highlight some of the digital health advances it prompted. We also describe emerging challenges and opportunities in the evolving ecosystem of digital health in the field of behavioral medicine, including the emergence of new evidence, research methods, and tools to promote health and health behaviors. Specifically, we offer updates on advanced research methods, the science of digital engagement, dissemination and implementation science, and artificial intelligence technologies, including examples of uses in healthcare and behavioral medicine. We also provide recommendations for next steps in these areas with attention to ethics, training, and accessibility considerations. The field of behavioral medicine has made meaningful advances since 2019 and continues to evolve with impressive pace and innovation.

## Introduction

For the 40th anniversary of the *Journal of Behavioral Medicine* (2019), we presented a “history and future” of digital health tools in behavioral medicine (Arigo et al., 2019). We outlined key successes, challenges, and opportunities with respect to technologies: wearable devices, mobile apps, and social media (see Table 1 for a summary of opportunities identified). We described the contributions of wearables, biofeedback, telehealth, social media platforms, and ambulatory assessment methods to the field of behavioral medicine. We highlighted the need for high-quality evidence, new research methods, an emphasis on ethics, and industry-academic collaboration. The science and practice of digital behavioral medicine have seen exponential and exciting growth over the past 5 years. Here we provide a narrative update on progress toward addressing the challenges we identified in 2019 and highlight recent advancements in these areas as well as in the emerging area of artificial intelligence.

**Table 1:** Updates on challenges to advancing digital health in behavioral medicine and areas for continued work

Challenge in 2019	Progress by 2024	Suggestions for future directions
Growing an evidence base for commercial devices/platforms	Growth in the commercial mobile health markets continues to accelerate and industry-academic partnerships continue to be key to success	Dissemination and increased use of pathways from industry-academic partnerships to commercialization of technologies
	Modest progress made in incorporation of BCTs in commercial apps Additional trials have also been tested to establish the efficacy of some commercial apps	Improved understanding about how to best use digital health tools to support BCTs in the context of interventions
Advances in research methods to support the complexity of digital health research questions	Expansion of research using factorial designs, SMART trials, N-of-1, micro-randomization, and hybrid designs have opened avenues to pursue more nuanced questions about how, when, and where to intervene and for whom	Research to capture real-time, contextualized effects of exposures and the use of skills promoted by digital tools (including between exposures)
	Advances in research on statistical methods and trials considerations for use of these designs	Ongoing work to disentangle between-person versus within-person effects of digital health tools and their components
Lack of a science of engagement	Advances in the conceptualization of both micro- and macro-engagement, including measurement of both	Research to understand the impacts of engagement as measured through objective versus subjective measures and the the impact of engagement quality on behavior change outcomes
	Expansion of research about the predictors of general engagement	Research to identify predictors of different types of engagement and testing of intervention strategies to facilitate them
	Emerging conceptualization of engagement as a dynamic process that may change over time in response to habit formation or other processes	Research to determine when and how often to measure engagement
Limited focus on principles versus technologies	Expansion of no-code platforms as a method to expedite app development	Exploration of the relative cost and time associated with development pathways to provide evidence of potential benefits of each
	Use of open APIs and large research consortia as methods to work collaboratively towards large-scale data collection and interoperability	Development of more open APIs to access health data for behavioral medicine tools, including use of APIs by researchers to provide access to the tools they develop

Sequential Multiple Assignment Randomization Trials (SMART); behavior change techniques (BCTs); application programming interfaces (APIs)

## Progress on 2019 challenges and opportunities

### New evidence supporting commercial devices/platforms

Since 2019, the use of commercial digital health apps and devices has continued to rise. The global digital health market was valued at $180 billion in 2023 and is expected to grow to $549 billion by 2028 (Markets & Markets, 2023). The Apple and Google Play stores now offer over 119,000 health apps (Kalinin, 2024). In 2023, 49% of US adults said they spent money on a health app in the past year (Bashir, 2024) and one third said they had used a wearable device (Dhingra et al., 2023). Behavioral medicine professionals have an opportunity to take the lead in communicating to the public which health apps and devices are grounded in good science.

In 2019, we noted that the use of evidence-based behavior change techniques (BCTs) in commercial health apps and devices was sparse. Recent reviews reveal some, albeit modest, improvement. For example, one review reported that asthma self-management apps included an average of 4 BCTs, with a range of 1–11 BCTs across apps (Ramsey et al., 2019); other reviews have found that physical activity apps for pregnant women (Hayman et al., 2021) and breast cancer survivors (Cooper et al., 2023) include 2–10 and 2–13 BCTs, respectively. Some popular commercial apps have added BCTs since they were initially reviewed in published articles, suggesting that frequent reviews may be necessary to capture the evolution of commercial products. Notably, in a 2013 review of commercial weight loss apps’ use of BCTs in the Diabetes Prevention Program (DPP) lifestyle intervention, the popular app Noom included only 25% of those BCTs (Pagoto et al., 2013). In 2017, Noom began offering human-delivered lifestyle coaching based on the DPP, which put Noom in full compliance with the DPP. In 2023 they launched Noom Med: this program pairs lifestyle coaching with GLP-1 receptor agonist medications, which produce weight loss that exceeds what can be achieved via lifestyle interventions alone (Noom, 2024). Other digital health companies are designing products using evidence-based behavioral intervention. For example, at least 6 commercial apps leverage cognitive behavioral therapy for insomnia (Erten Uyumaz et al., 2021) and several use evidence-based approaches to weight control such as the DASH diet (*n* = 7) and Mediterranean diet (*n* = 55; McAleese et al., 2022). These reviews offer important contributions by examining commercial health apps that were designed to disseminate evidence-based interventions.

Randomized controlled trials (RCTs) are the gold standard for establishing intervention efficacy and are increasingly being used to test commercial health apps, particularly those that already have large user bases. Such trials have established the efficacy of the apps Calm, Headspace, Noom, WW (WeightWatchers), and Talkspace on various clinical outcomes (Huberty et al., 2022; Song et al., 2023; Taylor et al., 2022; Thomas et al., 2017; Toro-Ramos et al., 2020). WeightWatchers is a notable example of a commercial program that has been tested in numerous clinical trials over the past 10 years as it has evolved from in-person delivery to hybrid (in-person and digital) (Ahern et al., 2017; Johnston et al., 2013) to digital only (Pagoto et al., 2023; Thomas et al., 2017). As we described in 2019, clinical trials for commercial products are often conducted via industry-academic partnerships, though many are conducted independently by academics. Researchers who do not have industry partnerships may consider the latter approach as an efficient alternative to attempting to develop new products. Given the sheer volume of commercial digital health products on the market, a great need exists for research on those that are already in the hands of millions of users. Research is also needed on the extent to which academia-produced digital health products reach the marketplace and attract users.

Some commercial tools are now routinely used in evidence-based behavioral interventions, given how effectively they assist users in enacting BCTs. For example, MyFitnessPal is a commercial calorie tracking app that is commonly used in weight control clinical trials to enable dietary self-monitoring and goal setting (e.g., Hoerster et al., 2020; Pagoto et al., 2022; Patel et al., 2020; Wang et al., 2017). Fitbit’s activity tracking devices, mobile apps, and scales are also used in clinical trials to enable self-monitoring (e.g., Miller et al., 2023). Further research is needed to determine how commercial digital health tools can be leveraged to support the execution of BCTs in interventions.

### Advances in research methods to support the complexity of digital health research questions

Traditional RCTs are the gold standard because they uniquely offer the advantages of random assignment to condition and thus, allow for drawing causal conclusions about the efficacy of interventions. RCTs are often inadequate and/or inefficient for evaluating digital health interventions, however. As we noted in 2019, behavioral interventions often provide “packages” of strategies to change behaviors that affect health outcomes. Unlike traditional face-to-face programs, the use of digital interventions is often self-guided, with respect to the frequency, sequence, and combination of exposure to various skills or components. Thus, in experimental conditions where participants receive the digital intervention, heterogeneity in users’ experiences of the intervention can make it impossible to draw strong conclusions about its effects. In trials that compare digital interventions to no intervention or to other interventions, this heterogeneity is often not reported, though it can mask meaningful effects for some participants. Thus, even when a digital intervention outperforms a comparator, we often don’t know the effective component(s), dose(s), sequence(s), or corresponding mechanism(s) of action.

New experimental designs can address these challenges. Factorial designs randomize a single participant to more than one condition across multiple factors at the start of treatment. As each participant “counts” toward more than one condition, this approach is efficient in that it maximizes power for tests of distinct components. Factorial designs may be uniquely useful for testing digital interventions, as they can efficiently test different combinations of components, doses, or sequences. In one ongoing trial, for example, Butryn and colleagues test the presence versus absence of sharing data from digital monitors of weight, dietary intake, and physical activity with 3 different support sources: the interventionist, a friend or family member, and a group of other participants in the trial (peers; Miller et al., 2023). As each participant is randomized to share ON versus OFF in each condition, it is possible to test the individual, additive, and synergistic effects of each type of data sharing. Factorial designs are now commonly used to optimize digital interventions using the Multiphase Optimization Strategy (MOST) framework (Collins & Guastaferro, 2021; Szeszulski & Guastaferro, 2024), an approach that has also seen impressive growth in popularity since 2019.

Sequential Multiple Assignment Randomization Trials (SMART) also use more than one randomization, but at different points in treatment, to test *dynamic treatment regimens* (Kidwell, 2015). This can help us understand how to proceed at specific decision points in treatment, such as when patients do not respond to the initial package of skills. With SMART, participants are re-randomized if they meet prespecified criteria at the decision point (or at multiple points), to determine the best overall approach to treatment for different participant trajectories. For example, an ongoing SMART by Zhao et al. (2022) tests whether adding personalized text messages improves smoking cessation rates among non-responders to generic messages, and whether adding other digital components can improve cessation rates among non-responders to personalized text messages. Many trials that use this design are not yet complete and their impact is yet to be determined. Yet, there is clear interest in the promise of SMARTs to generate personalized digital interventions.

N-of-1 and micro-randomization can reveal the effects of digital health tools as they are being used, *within-person* (cf. Walton et al., 2018). Most trial designs compare people to each other with respect to treatment response, aggregating across weeks, months, or years to determine a single estimate of response for each person in an assigned condition. The resulting information about *who* experiences long-term change is invaluable. But behavioral treatments are intended to induce a set of changes in cognitions, emotions, and behaviors through practice in daily life; we don’t yet know much about the translation of skills taught during intervention exposures to behavior change between exposures in daily life, because we don’t use methods that can appropriately assess these processes. Worse, although digital tools are meant to reach participants wherever they are, in their natural environments, we rarely capture what happens *when* participants are exposed to digital components or the differences in a given person’s response to different components (in the moment or in the near future).

This is a missed opportunity that may have negative consequences for health. For example, although exposure to social media has shown a negative association with well-being between-person, there is little (if any) within-person association (Stavrova & Denissen, 2021). Moreover, among adolescents, although greater social media use is positively associated with relationship well-being between-person, it is *negatively* associated with well-being within-person (Pouwels et al., 2021). Similar divergence may exist for common behavior change techniques in digital interventions (e.g., activating social comparison processes via leaderboards; Arigo et al., 2020), though our methods overlook them. Disentangling these associations is critical to improving the effectiveness of digital tools for health behavior change. Intensive longitudinal assessment designs such as ecological momentary assessment and ambulatory daily diaries capture psychological experiences and behaviors as they occur in real time, using technologies such as smartphones and wearables (Smyth et al., 2017). These designs are increasingly popular and can be combined with experimental methods (e.g., micro-randomization) to test the immediate and short-term effects of digital components on their purported mechanisms of action and longer-term outcomes (Arigo et al., 2024).

Although advanced experimental and intensive research designs existed in 2019 and we referenced them in our earlier paper, databases showed that very little research was published and funding agencies listed very few funded grants using these designs. Fortunately, we have made considerable progress since 2019: PubMed, Google Scholar, and NIH Reporter now contain multiple pages of studies using these designs for tests of digital tools to support physical activity, weight management, smoking and substance use cessation, oral health care, and medical decision making, as well as to improve *engagement* with digital resources that support these behavior changes. Recent developments provide even more reason for optimism. These include published guidance on statistical methods for advanced trial designs (Cohn et al., 2023; Montoya et al., 2023; Yeaton, 2024), as well as guidance on the advantages, design considerations, and evaluation of these trials that are written for diverse audiences (e.g., SMART; Kidwell & Amirall, 2023). Finally, *hybrid experimental designs* have been developed to address complex hypotheses about both human-delivered and digital components of an intervention (Nahum-Shani et al., 2022). Specifically, these designs can combine traditional group-level randomization (single or multiple randomizations) and intensive longitudinal assessment to understand the effects of intervention packages at different timescales (Nahum-Shani et al., 2024). Such designs offer unique opportunities to determine when and how exposure to intervention components leads to behavior change *between* exposures, elucidating the pathways linking digital interventions to health outcomes.

### Advances in the science of engagement

The utility of digital behavior change interventions rests on their ability to engage users. Many studies show that greater user engagement predicts better outcomes (Donkin et al., 2011; Lehmann et al., 2024; Power et al., 2019), though promoting engagement can be challenging. It is also measured in highly variable ways and we know little about how much engagement is optimal (Nahum-Shani & Yoon, 2024). Conceptual frameworks that define engagement and its measurement are emerging to address this need (Nahum-Shani & Yoon, 2024). Broadly, researchers must consider what (specifically) users engage with and how they engage. In terms of *what*, engagement can be thought of as either micro-engagement (i.e., “little e”) or macro-engagement (i.e., “big E”; Cole-Lewis et al., 2019). Micro-engagement refers to user engagement with the intervention interface (e.g., clicks, pages visited) and/or technology-facilitated behavior change strategies (e.g., self- monitoring), whereas macro-engagement refers to user engagement in the target health behavior (e.g., physical activity; Cole-Lewis et al., 2019).

Emerging frameworks for micro-engagement in digital behavior change interventions propose that engagement is multifaceted, including behavioral, cognitive, and affective components. Specifically, Perski et al. (2017) operationalize engagement using objective (e.g., duration of use) and subjective (e.g., perceived interest) measures. Objective measures are more commonly used and typically involve assessing the frequency (i.e., number of uses), intensity (i.e., amount of behavior recorded, such as the number of diet logs or exercises), time spent using the technology and each feature, and type of engagement (Bijkerk et al., 2023). Subjective measures are newer and typically employ self-report via surveys and/or qualitative interviews (Kelders et al., 2020a, 2020b). Engagement has also been conceptualized as active, referring to any engagement in which the user is interacting with the technology, or passive, referring to the user consuming information from the technology but not interacting with it (Perski et al., 2017). Of note, an underexplored aspect is *quality*, or the degree to which a user engages with the technology as intended (Bijkerk et al., 2023). Little is known about how these affect behavior change.

Objective measures such as frequency of use are important to include in efficacy trials of digital health tools, but such measures cannot always be compared across different technologies. For this reason, 2 self-report measures have been developed since 2019 that assess engagement across a wide range of digital behavior change interventions. The Digital Behavior Change Intervention (DCBI) Engagement Scale (Perski et al., 2020) uses 10 items to capture the user’s reported amount and depth of use, interest, attention, and enjoyment. Scores on this scale were not associated with objective measures of current or future use of the technology, but the extent to which objective and subjective measures of engagement should be related is unclear. Building on this work, Kelders et al. (2020a) developed the Twente Engagement with eHealth Technologies Scale (TWEETS), a 9-item measure of behavioral (e.g., “This technology is part of my daily routine”), cognitive (e.g., “This technology makes it easier for me to work on my goal”), and affective (e.g., “I enjoy using this technology”) engagement with a digital tool. Scores on this scale are associated with scores on the DCBI Engagement Scale and overall perceived behavior change, but not with self-reported frequency of technology use. Because self-report measures capture subjective engagement, using them alongside objective measures of engagement with the technology and the target behavior might be the most comprehensive approach.

Research on predictors of engagement with digital health tools may also be useful for increasing our understanding of engagement. Predictors of high engagement include motivation, self-efficacy, expectations, personal relevance of the technology, receipt of social support via the technology, access to human-delivered counseling, novelty, personalization, aesthetically-pleasing design features, and credibility of the technology predictors of poor engagement include stress, depression, greater symptom severity, and limited access to healthcare (Bijkerk et al., 2023; Perski et al., 2017). Additional work is needed to identify predictors of different facets of engagement (e.g., behavioral, cognitive, affective) and to test intervention strategies that facilitate engagement. For example, a recent meta-analysis of strategies to improve engagement in obesity interventions revealed that social support, shaping knowledge, repetition and substitution, natural consequences, and email or text messages improved engagement (Grady et al., 2023). However, engagement was typically defined narrowly (i.e., frequency of use of the technology), with only 54% of studies using subjective measures of engagement.

Nahum-Shani and Yoon (2024) propose that digital interventions be conceptualized as a collection of stimuli and tasks that may be digital or non-digital, and that engagement can be considered a process of evolving reactions to those stimuli and tasks. As an example of this evolution, high engagement may facilitate habit formation and to the extent that this occurs, engagement with digital stimuli may eventually decline because stimuli are no longer needed to cue the behavior. On the other hand, declining engagement with digital stimuli over time may signal habituation, intervention burden, or other barriers to engagement, and thus, poor outcomes. Conceptualizing engagement as a process may shed light on mechanisms by which engagement influences outcomes in both positive and negative ways.

Although the science of engagement is growing, many questions remain. For example, little is known about when and how often to measure engagement during an intervention. Engagement is dynamic such that it varies over the course of an intervention based on contextual factors, experience with the intervention, and need for further intervention (Bijkerk et al., 2023). Frequent assessment of engagement can be used to identify points during the intervention when users are most likely to disengage. Dynamic or adaptive interventions may then be useful in providing additional intervention to users *before* they are likely to disengage. Research is also needed to understand how distinct facets of engagement (e.g., behavioral, cognitive, affective) change over time and how different trajectories of engagement are related to behavior change and health outcomes. In addition, it is imperative to better understand which intervention components influence different types of engagement (Milne-Ives et al., 2023). The answers to these questions may differ based on the intervention type, target behaviors, and target population. As such, progress on the science of engagement will require researchers to use advanced research methods and comparable measures and metrics of engagement across studies.

### Progress toward a focus on principles versus technologies

In 2019, we noted that technology evolution far outpaces research on these tools, and that tools developed by researchers rarely reach the commercial market. While these problems persist, we have made progress with respect to innovative approaches for efficient and scalable mobile app development. The first is “no-code” platforms, which use intuitive drag-and-drop formatting and pre-built module components; thus, researchers do not need extensive training or a programming background to build app prototypes (Liu et al., in press). Early studies using no-code platforms included the development of an app to reduce sedentary time (Bond et al., 2014). A recent scoping review of no-code tools used to design physical activity apps found 11 platforms to date (e.g., Avicenna, Expiwell, LifeData; Liu et al., in press). Of these, 8 were available with both iOS and Android versions and 7 had multilanguage support. Direct cost analysis and time comparison between no-code and traditional development methods are needed to determine the full potential for no-code platforms.

A second innovation is in open application programming interfaces (APIs) that allow for access without custom programming. For example, the Substitutable Medical Apps and Reusable Technology Health IT project facilitates connection to electronic health record systems. Funded by the U.S. Office of the National Coordinator of Health Information Technology, this project has supported the development of over 100 apps (Smarthealth IT, 2024). Associated federal regulations now require that all electronic health records systems in the U.S. embed two APIs to provide standardized access to health data across systems, ensuring interoperability (Mandl et al., 2024). This broad national approach provides a powerful infrastructure for data accessibility and app development through APIs. In behavioral medicine, open APIs are less common, but are offered through select commercial health apps, helping to facilitate participant data access (see Fitbit, 2024; Fatsecret, 2024). For example, a researcher can develop an app to deliver a behavior change intervention in conjunction with providing a Fitbit, such that participant data collected by a Fitbit device (e.g., physical activity, sleep) are seamlessly integrated. Researchers and commercial health app developers should consider the potential for expanded use of development of standardized systems and APIs that allow for expedited app development and data exchange in the development of future technologies.

A third innovation is the establishment of large research consortia that are unified around common goals of collecting similar outcomes data across studies. Similar to the use of large-scale APIs, this approach has not yet been applied fully to behavioral outcomes but stands as a potential model. An example is the Remote Assessment of Disease and Relapse—Central Nervous System (RADAR-CNS), developed in Europe to support remote monitoring of major depressive disorder, epilepsy, and multiple sclerosis using wearable devices and smartphone technology (Ranjan et al., 2019). The program was funded by the Innovative Medicines Initiative (a public–private partnership between the European Federation of Pharmaceutical Industries and Associations and the European Union; King’s College London, 2024). The platform supported the recruitment of 1,450 participants and collection of 62 terabytes of data, which were recently released under an open-source license to promote use by the broader community. Overall, innovative approaches to the development and efficient scale-up of mobile technologies are increasingly available but underutilized, given their potential.

## New frontiers, challenges, and opportunities for digital health in behavioral medicine

### Dissemination & implementation science and technology scalability

Despite the development of countless digital health technologies by researchers, few have made the leap from research to broad use at scale. The next era of digital health interventions should leverage commercialization opportunities, as well as dissemination and implementation (D&I) science to ensure adoption in real-world settings. D&I science offers 2 key directions for digital health research. First, it provides frameworks by which to consider which existing technologies with proven efficacy should be used in real-world settings (e.g., healthcare, education, employer systems) and how to evaluate implementation in those settings, with consideration of the multilevel factors that make them more or less likely to be adopted (Shelton et al., 2020). Second, they challenge researchers to consider the context in which tools will be used and the resources available in those contexts, suggesting that future technologies should be designed for dissemination and real-world implementation from the start (see Table 2).

**Table 2:** New frontiers, challenges, and opportunities for digital health in behavioral medicine

New frontiers and challenges	Key points	Suggestions for future directions
D&I science and technology scalability	Most digital interventions are difficult to deliver at scale and are rarely commercialized, as there are barriers to integration with existing systems D&I represent distinct disciplines focused on the translation of efficacious approaches to real-world settings	Emphasis on scalability, dissemination, and implementation concerns from the development stage Use of tools such as no-code platforms and APIs to promote interoperability and facilitate broader use
Remote data collection and telehealth	Collecting data and delivering treatment remotely increases accessibility, reduces transmission of infectious diseases, and shows similar treatment outcomes to in-person approaches	Additional work to establish best practices for remote data collection protocols and high-quality evidence for the efficacy of remote intervention Advocacy for the continued reimbursement of remote treatment delivery and parity with in-person services
AI	Tools such as machine learning and large language models have been leveraged to summarize large datasets, generate intervention content, and provide two-way patient education	Continued exploration of opportunities to leverage these technologies in behavioral medicine
Ethical considerations	Privacy, data security, and health equity continue to present challenges, in part due to shifting legal landscapes and identification of biases in digital systems	Increased attention to these issues, the limitations they present for behavioral medicine, and opportunities they present for behavioral medicine professionals to lead improvements Wider use of tools such as the Digital Health Checklist (ReCODE Health)
Training and collaboration	Despite the availability of new, complex digital technologies and advanced research methods to generate needed evidence, behavioral medicine professionals rarely have support for staying up to date in these areas	Emphasis on digital technologies, D&I and ethical concerns, and advanced research methods in behavioral medicine training programs Greater availability of continuing education resources and the protected time to use them Frequent and close collaboration between professionals with and without expertise in these areas

Dissemination & Implementation (D&I); Artificial Intelligence (AI)

To date, few trials have focused on the implementation of digital health tools in real-world settings. A notable exception is the US Veterans Health Administration, where myriad digital tools have been adopted and routinely implemented for a variety of health issues (e.g., smoking cessation, weight management; Blok et al., 2019; US Department of Veterans Affairs, 2024). The VA’s centralized electronic health record system and payment structures across this clinical context have greatly facilitated research and implementation (Jackson et al., 2011). However, despite the wide availability of health apps specifically for veterans, a recent survey found that uptake is low and the strongest predictor of veteran use of VA-created apps is provider encouragement, which results in nearly 3 times higher odds of use (Hogan et al., 2022). Thus, even in a large, established, and digitally integrated health system, health app uptake in routine practice is low. Additional research and support is are needed to encourage providers to prescribe these tools to patients.

Beyond the VA, implementation of digital health tools in real-world settings has been sparse. Notable examples include a study that examined implementation of digital referrals to web-assisted tobacco interventions in community-based primary care practices, which found that digital referrals produced similar referral rates to a paper system, but threefold greater conversion to intervention registrations (Houston et al., 2015). Implementation facilitators included ease of using the system and the perceived intervention efficacy (Houston et al., 2015). Similarly, the Home BP trial tested a digital intervention for hypertension management in primary care. Researchers first undertook a systematic intervention planning process to consider multilevel factors impacting potential implementation, including feedback from patients and health professionals. In a subsequent randomized trial, they tested their digital intervention versus usual care and collected implementation data (e.g., cost effectiveness) to inform clinical rollout, finding that the intervention led to better hypertension management than usual care and with minimal incremental costs (McManus et al., 2021). Here too, more research is needed on barriers and facilitators to implementation of digital tools in routine practice.

Implementation trials that focus solely on testing strategies to implement digital tools in existing settings and structures are also needed. One such study is the ongoing DIGITIS Trial which tests strategies to integrate prescription digital therapeutics for substance use disorders (Glass et al., 2023). Using a factorial design, clinics are randomized to receive different combinations of implementation techniques to identify the optimal overall approach. Additional insights to facilitate implementation, particularly its sustainability in the clinical setting, will come from the extensive ongoing work with digital mental health treatments (Meyerhoff et al., 2023; Mohr et al., 2021). For example, an interdisciplinary international group of healthcare experts convened in 2019 to consider the barriers and facilitators to broad adoption of digital mental health tools (Mohr et al., 2021). They found that while there is consensus that the tools are effective and cost-effective, there are still complications with proper reimbursements and there is not an established way to evaluate the tools, preventing further clinical implementation (Mohr et al., 2021).

Yet another limit to the implementation of digital behavioral medicine tools is that the quality of evidence thus far has not been strong enough to move many digital solutions to clinical application. For example, a recent review identified 721 studies that describe virtual reality technologies for mental health, yet weaknesses in study design have hindered progression toward clinical adoption (Wiebe et al., 2022). Specifically, few studies use rigorous and evidence-based processes at both the technology development and initial clinical testing phases, resulting in data that cannot support broad implementation (Selaskowski et al., 2024). A protocol-based dual publication model, which is similar to a registered report but specific to the development and evaluation of digital technologies for clinical application, has been proposed to improve methodological quality (Selaskowski et al., 2024). This is a promising approach, as it would encourage more detailed description of the technology development methods and foster greater replicability of digital tools while requiring robust clinical studies to demonstrate the efficacy data needed to justify further use and testing.

### Remote data collection and telehealth

The COVID-19 pandemic accelerated a shift to remote data collection and treatment delivery in an effort to reduce the transmission of infectious disease. Remote options are also more accessible than in-person approaches and may offer needed flexibility for hard-to-reach and underprivileged groups, as the burdens of transportation and childcare are minimized or removed altogether. Remote methods typically leverage Bluetooth or wireless connected devices, wearable devices, mobile apps, online platforms, and/or video teleconferencing software. Some of these are freely available (e.g., Zoom) and as noted, some are already in widespread use (e.g., Fitbit), and evidence to support the validity of remote methods is growing. Specifically, evidence shows that weight, waist circumference, and movement assessments can be conducted with cancer survivors (Hoenemeyer et al., 2022), older adults (Villar et al., 2024), and veterans (Ogawa et al., 2021) via Zoom video call, with high reliability and high concurrence with in-person methods. Similar trials are in progress to assess the validity of remote assessments of physical performance and mobility among older adult cancer survivors (Blair et al., 2020).

For those who do not already use these technologies, however, remote protocols may be expensive for researchers and clinics, and poor execution may result in suboptimal patient engagement. For example, Hoenemeyer et al. (2022) note that high shipping costs for the equipment necessary to conduct remote arm curl and grip strength tests (i.e., mailing dumbbells to participants’ homes) prevented the team from including these typical tests in their remote trial. Even when technology or equipment are available (e.g., Zoom), technical difficulties such as poor internet connectivity and environmental conditions such as poor lighting, incorrect camera angles, and distractions in the home can result in low engagement and lower-quality data, relative to in-person procedures. Participants may also perceive researchers to be less directly engaged in remote meetings than in person, as researchers often have to manage multiple tasks simultaneously (e.g., screenshare, recording responses; McClelland et al., 2024).

Studies conducted during the transition from in-person to remote treatment during the pandemic revealed additional challenges, such as declines in use of behavioral strategies such as self-monitoring (Bernhart et al., 2022) and suboptimal acceptability of remote procedures (at least initially) in certain subgroups (e.g., older adults; Pisu et al., 2021; Ross et al., 2021), possibly due to low technology literacy. Recommendations to address such barriers include encouraging participants to have cameras on during video calls (to promote attention and engagement), training research staff to look at the camera rather than the screen (for more direct eye contact) and limit distractions such as electronic notifications, and encouraging both participants and research staff to log into meetings early to troubleshoot any technical problems (McClelland et al., 2024). Researchers can also consider building in fallback options such as phone calls (if technical difficulties cannot be resolved) and offering breaks during longer sessions (McClelland et al., 2024), and build these options into protocols from the start (rather than as deviations from the expected protocol).

Yet, recent evidence for treatment outcomes is highly encouraging: in 2 trials that pivoted from in-person to videoconference-delivered behavioral weight loss intervention, weight loss was comparable between groups who received hybrid in-person (pre-pandemic) followed by remote (during pandemic) and remote-only treatment (Ross et al., 2022; Tchang et al., 2022). Similarly, studies show little (if any) difference between in-person and remote (telehealth) treatment for mental health outcomes (Bulkes et al., 2022; Lin et al., 2022), and attrition does not differ between modalities (Giovanetti et al., 2022). Thus, research increasingly demonstrates that fully remote treatment protocols can produce meaningful change in clinical outcomes with the potential for less burden on participants and patients.

Remotely delivered telehealth services were available before the COVID-19 pandemic, though adoption in routine clinical practice occurred mostly in rural and other settings where access was limited, and reimbursement policies varied greatly across states (Brotman & Kotloff, 2021). Reimbursement barriers were lifted during the pandemic to address the critical public health need. Federal programs such as Medicare have maintained reimbursement for telehealth services through 2024 (Department of Health & Human Services, 2024), but the future is uncertain: some insurance providers offer lower financial compensation for telehealth than in-person visits (Aremu et al., 2022) and the sustainability of legislative and budgetary support for telehealth is unclear. The field of behavioral medicine should prioritize establishing the efficacy for remotely delivered interventions given that such data are needed to inform reimbursement policy, and should continue to advocate for telehealth reimbursement.

### Artificial intelligence (AI)

AI, or “technology that simulates human intelligence and problem-solving capabilities” (Stryker & Kavlakoglu, 2024), is increasingly used in daily life and healthcare (e.g., GPS, digital assistants, social media algorithms, ChatGPT) and is a relatively new frontier for behavioral medicine. AI has myriad applications in behavioral medicine and has the potential to improve measurement and prediction of behavior and clinical outcomes with far greater precision than traditional methods (Bucher et al., 2024). AI also has potential to help us design more personalized and effective interventions, which are urgently needed given the rapid evolution of data sources during the 21st century. Traditional data sources have included biological assays, surveys, and focus group/interviews, but in recent years, intensive longitudinal assessment methods, wearable devices, mobile applications, and online platforms have been used to collect high volumes of data (i.e., big data). AI is well-suited for handling big data and its use offers new ways to understand, predict, and intervene on health behavior. Machine learning, natural language processing, generative AI and large language models, and computer vision are four types of AI methods that have great potential to revolutionize behavioral medicine research.

*Machine learning* (ML) uses algorithms that learn from data to make predictions, identify patterns, and/or make decisions (Shalev-Shwartz & Ben-Davd, 2014). Applications in healthcare include predicting patient outcomes, risk for disease, and disease outbreaks; creating tailored treatment plans; and improving the efficiency of healthcare systems (Dixon et al., 2024). In behavioral medicine, ML has been used to predict intervention outcomes (Khalilnejad et al., 2024), diet lapses during weight loss treatment (Goldstein et al., 2018), smoking behavior (Yu et al., 2024), patient adherence (Masiero et al., 2024), and depressive symptoms (De la Barrera et al., 2024). ML has also been used to predict behavior in real time and to optimize and personalize behavioral interventions (Forman et al., 2019; Presseller et al., 2023; Rocha et al., 2023; Scodari et al., 2023). *Natural language processing* (NLP) uses algorithms to understand, process, and analyze human language (AlShehri et al., 2024; Vaniukov, 2024), and is leveraged in conversational agents and large language models. NLP has been used in medicine to analyze speech and text from a range of sources including clinic notes, patient comments in an electronic health record, and the research literature to aid in diagnosis, prevention, and patient engagement (AlShehri et al., 2024; Petti et al., 2020). Behavioral medicine researchers use NLP to study qualitative and/or social media data (Cha & Lee, 2024; Lau et al., 2024; Patra et al., 2023), assist with dietary self-monitoring via voice or text entry (Chikwetu et al., 2023), and identify patient-reported outcomes via clinical notes in electronic health records (Ebrahimi et al., 2024; Sim et al., 2023).

*Generative AI (genAI)* is the use of AI to generate content of all types (e.g., text, images). Large language models (LLM) are a form of genAI that produces text (Yu et al., 2023). LLMs perform tasks in response to text queries and generate human-like responses via a computer program that has been trained on vast amounts of data to interpret human language. They can generate content, engage in conversations, and answer questions, and they are used in conversational agents, text analysis, code generation, language translation, and text summarization (Thirunavukarasu et al., 2023). Conversational agents, a popular type of LLM, use ML and NLP to engage in conversations with humans (Laranjo et al., 2018). Chatbots are one type of conversational agent in which a software program automates specific conversational tasks, like answering a finite set of questions or providing information or assistance to a user (Tudor Car et al., 2020). In behavioral medicine, chatbots have been used for weight management, vaccine communication, smoking cessation, and chronic disease management (Aggarwal et al., 2023; Bak & Chin, 2024; Noh et al., 2023; Passanante et al., 2023). Conversational agents can be used to counsel and educate patients about these and other topics, which may reduce the intensity of behavioral interventions that rely on human delivery.

ChatGPT, often used as a conversational agent, is perhaps the most notable LLM, as it exploded in popularity after its release by OpenAI in 2023 (OpenAI, 2023). In medicine, ChatGPT and other LLMs assist with clinical notes and summaries, answer medical exam or patient questions, provide patient education, augment medical training (Omiye et al., 2024), conduct cancer screening and genetic counseling, assess symptoms, and support caregivers (Jiang et al., 2024). In behavioral medicine, researchers have examined the accuracy of LLMs in providing patient education (Kozaily et al., 2024), debunking health misinformation, developing exercise programs, recommending evidence-based treatments, identifying motivational states, and even conducting systematic reviews (Amin et al., 2023). Behavioral medicine researchers are also using LLMs to create intervention content (Willms et al., in press).

*Computer vision* may also have myriad uses in behavioral medicine, as it uses videos and/or images to train a computer program to recognize and interpret visual content. These data are gathered via sensors and/or cameras and ML algorithms perform object detection and image classification. For example, computer vision has been used to diagnose skin cancer by detecting characteristics of skin lesions not visible to the naked eye (Akilandasowmya et al., 2024), to classify different types of back pain based on movements (Hartley et al., 2024), to detect pain and acute patient deterioration via changes in facial expression, to monitor mobility in intensive care patients, to detect falls in the elderly, and to assist in the diagnosis of autism via head motion and facial expression data (Lindroth et al., 2024). In behavioral medicine, computer vision has been used to identify foods based on pictures taken by users and use this information to tailor intervention messages (Chew et al., 2024). Together, AI tools have enormous potential to increase the efficiency, accuracy, and reach of behavioral medicine interventions, making this an exciting new area for growth.

Although AI has extraordinary potential to improve individual and public health, it also has extraordinary potential to negatively impact health. For example, AI can be used to produce deepfakes and large volumes of disinformation, in the forms of authentic-appearing online articles with fictional medical references, social media posts and comments, and patient and physician testimonials (Menz et al., 2024). The implications of the ability to rapidly develop and disseminate disinformation are likely to be far-reaching and evidence suggests that AI-powered disinformation campaigns are already infiltrating low- and middle-income nations (Hotez, 2024). In the past few years, the World Health Organization (WHO) has issued statements urging the judicious use of AI for health (World Health Organization, 2023) and laid out guidance on the ethical use of AI for health (World Health Organization, 2021). However, the WHO’s very own health chatbot, S.A.R.A.H. (Smart AI Resource Assistant for Health; World Health Organization, 2024) has come under fire for providing outdated information (Nix, 2024) and includes a disclaimer stating that “answers may not always be accurate” (World Health Organization, 2024). The WHO is calling for researchers to help them identify ways their chatbot can be used to disseminate accurate health information (World Health Organization, 2024). Given the chatbot’s focus on healthy lifestyle topics such as quitting smoking, physical activity, healthy diet, and stress reduction, behavioral medicine researchers are well-positioned to lead this charge. Generally, much research is needed on both the benefits and dangers of genAI to public health.

### Ethical considerations

In 2019 we called attention to issues of privacy and data security, to promote the responsible use of digital health tools in behavioral medicine research and practice. Groups such as ReCODE Health (2024) provide a wealth of resources on this topic, including the Digital Health Checklist (Nebeker et al., 2021), which provides guidance to researchers and ethics committees with respect to the use of digital tools in behavioral research. Such resources are invaluable and support for their ongoing revision is essential as the digital health landscape continues to evolve. An important example comes from the 2022 US Supreme Court decision *Dobbs v. Jackson Women’s Health Organization*, which overturned *Roe v. Wade*. This decision had immediate implications for digital health. With states moving to outlaw abortion and related reproductive healthcare, data from self-monitoring tools such as Fitbit and menstrual cycle tracking apps could be subpoenaed by law enforcement to aid in the prosecution of those who perform and/or receive restricted services (Kim, 2022). In the wake of announcements that companies would make such data available, many users of these tools reported concerns about their use in research; some indicated that they would decline to participate in such research if use of these tools were required, particularly users who identified with minoritized groups (Salvatore et al., 2024). Legislation restricting reproductive healthcare presents new ethical issues in women’s health research, and protecting the privacy and security of digital health data in behavioral medicine research is paramount.

The use of AI also comes with ethical considerations related to bias, privacy, and informed consent. Algorithmic bias can occur when data used to train AI are biased, when the AI is used in a different context or population than the one for which it was originally designed, or when the AI’s results are interpreted in a biased way (National Library of Medicine, 2024). Such biases may widen health inequities rather than help to close them. The Agency for Healthcare Research and Quality and the National Institute for Minority Health and Health Disparities recommend the following principles to reduce bias in AI: (1) promote health equity during all phases of the algorithm life cycle, (2) ensure algorithms and their use are transparent and explainable, (3) engage patients and communities during all phases, (4) identify algorithm fairness issues, and (5) establish accountability for equity and fairness in outcomes emanating from algorithms (Chin et al., 2023).

Privacy is another ethical consideration in the use of AI, given the risk that protected health information ends up being used to train algorithms. AI uses must be HIPPA-compliant and privacy protections must be built-in and resistant to data breaches. In 2023, the American Psychiatric Association issued an advisory to clinicians and researchers against the use of patient information in AI systems (American Psychiatric Association, 2023). Researchers must be transparent about the data being used in AI systems and its potential biases and be mindful about informed consent, which can only be obtained when researchers can explain the technology used, how patient data will be used, and the potential limitations and biases of the technology (Diaz-Asper et al., 2024). This is also relevant in clinical settings when AI is used for diagnosis and/or treatment decision making (Park, 2024). Because AI evolves faster than ethical best practices can be established, research is needed on potential harms and ethical issues emanating from the use of AI in behavioral medicine research and practice.

### Training and collaboration

To continue to advance behavioral medicine via the use of digital tools, and to ensure high ethical standards and dissemination, 2 efforts are critical: ensuring that training curricula stay up to date and fostering expert consultation and collaboration. For training programs, emerging digital technologies and research methods that can address complex research questions must be included in standard curricula. This will not only ensure that trainees use these tools and designs in their work, but that they will be equipped to train future generations. For example, behavioral medicine training programs should include emphasis on advanced research methods and AI and include specific skill sets such as machine learning and prompt engineering (i.e., crafting generative AI prompts that produce high-quality output; Amazon Web Services, 2024). Continuing education for researchers who have not received such training is also needed and professionals need the protected time and resources to take advantage of such training opportunities. For researchers interested in developing digital health tools, we strongly recommend seeking out training in commercialization and entrepreneurialism, to learn how to bring a product to market and thereby maximize the resources invested in development. Efforts are also needed to educate researchers about alternative development pathways such as no-code platforms. Finally, the need for transdisciplinary teams has never been greater; teams benefit from representing and integrating expertise from behavioral science, computer science, human–computer interaction, and technology ethics.

## Conclusion

Digital health tools present exciting opportunities to revolutionize how we conceptualize, study, and intervene on health behavior. The field of behavioral medicine has made impressive advances since 2019, clearly capitalizing on these opportunities. As digital technologies continue to evolve, the field needs to keep pace so we can continue to offer our unique expertise in the broad landscape of healthcare and public health. This requires specific attention to research methods, engagement, remote protocols and services, D&I efforts, and ethics, as well as to training and collaboration. The next 5 years are likely to involve increasing use of advanced research designs, digital health tools, and AI in the field of behavioral medicine. This increase has the potential to accelerate our progress toward the development and testing of more effective, engaging, and personalized interventions that have high potential for dissemination and implementation.

Note: Methods such as the Design Sprint process, a 5-day exercise based on agile and user-centered design principles, can accelerate development and implementation regardless of the technology selected. We recommend the following resources for more information about this process:

- Knapp, J., Zeratsky, J., & Kowitz, B. (2016). *Sprint: How to Solve Big Problems and Test New Ideas in Just Five Days*. Simon and Schuster.
- Jake-Schoffman, D.E., & McVay, M.A. (2021). Using the Design Sprint process to enhance and accelerate behavioral medicine progress: a case study and guidance, *Translational Behavioral Medicine, 11*(5), 1099–1106, https://doi.org/10.1093/tbm/ibaa100
